# Baltimore College of Dental Surgery

**Published:** 1846-05-01

**Authors:** 


*Baltimore College of Dental Surgery.*—This institution appears to be in a highly flourishing condition. At its recent commencement diplomas were conferred upon nine graduates. Prof. C. A. Harris delivered a valedictory address on the occasion which was happily responded to by one of the graduates. We are indebted to Prof. C. for a copy of his address, which has been published. It is replete with worthy sentiments and wholesome counsel.

